# High Glucose Promotes and Aggravates the Senescence and Dysfunction of Vascular Endothelial Cells in Women with Hyperglycemia in Pregnancy

**DOI:** 10.3390/biom14030329

**Published:** 2024-03-10

**Authors:** Lin Zheng, Mingqing Li, Huaping Li

**Affiliations:** 1 Department of Gynecology and Obstetrics, Shanghai Sixth People’s Hospital Affiliated to Shanghai Jiao Tong University School of Medicine, Shanghai 200233, China; 121725910639@sjtu.edu.cn; 2 Laboratory for Reproductive Immunology, Shanghai Key Laboratory of Female Reproductive Endocrine Related Diseases, Obstetrics and Gynecology Hospital of Fudan University, Shanghai 200080, China; 3 Department of Gynecology and Obstetrics, Jinshan Branch of Shanghai Sixth People’s Hospital, Shanghai 201599, China

**Keywords:** hyperglycemia, pregnancy, cellular senescence, umbilical vein endothelial cells

## Abstract

Hyperglycemia in pregnancy (HIP) is linked to fetoplacental endothelial dysfunction, which might be a result of hyperglycemia. Hyperglycemia is associated with cell senescence; however, the role and mechanism of high glucose and cell senescence in HIP endothelial cell failure are largely unknown. Our study discovered that human umbilical vein endothelial cells (HUVECs) obtained from HIP pregnant women exhibit excessive senescence, with significantly elevated expression of senescence markers senescence-associated beta-galactosidase (SA-β-gal), p16, p21, and p53. Subsequently, we found that exposing primary HUVECs and cell lines to high glucose resulted in an increase in the synthesis of these senescence indicators, similar to what had been observed in pregnant women with HIP. A replicate senescence model and stress-induced premature senescence (SIPS) model showed higher amounts of vascular damage indicators, including von Willebrand factor (vWF), chemotactic C-C motif chemokine ligand 2 (CCL2), intercellular adhesion molecule 1 (ICAM-1), along with the anti-apoptotic protein BCL2. However, lower expressions of the pro-apoptotic component BAX, in addition to defective proliferation and tubulogenesis, were seen. Further studies indicated that hyperglycemia can not only induce these alterations in HUVECs but also exacerbate the aforementioned changes in both aging HUVECs. The experiments outlined above have also been validated in pregnant women with HIP. Collectively, these data suggest that exposure to high glucose accelerates cell senescence-mediated vein endothelial cell dysfunction, including excessive inflammation, cell adhesion, impaired angiogenesis, and cell proliferation possibly contributing to pregnancy complications and adverse pregnancy outcomes.

## 1. Introduction

Globally, diabetes significantly contributes to mortality and is a contributing factor to severe negative consequences and fatalities among COVID-19 patients. Hyperglycemia in pregnancy (HIP) is a special type of diabetes, which is often categorized as pre-gestational diabetes, diabetes in pregnancy (DIP), or gestational diabetes mellitus (GDM) [1,2]. The tenth version of the annual IDF Diabetes Atlas [3], developed by the International Diabetes Federation (IDF), reported that the worldwide occurrence of HIP in 2021 would reach up to 16.7%, impacting roughly 21.1 million live births and affecting approximately one in six pregnancies. HIP is known to lead to various complications and negative pregnancy results, including but not limited to preterm birth (PTB), hypertensive disorders of pregnancy (HDP), intrauterine growth restriction, and respiratory distress syndrome, among others. However, the mechanism behind hyperglycemia in pregnancy and its adverse pregnancy outcomes remains unclear.

Placental dysangiogenesis is a major cause of numerous pregnancy problems and poor pregnancy outcomes [4]. The activity of vascular endothelial cells might be hampered as a result of hyperglycemia in various ways [5]. Recent research has revealed that endothelial cells in the umbilical vein of HIP pregnant women exhibit various endothelial cell dysfunctions [6], including elevated levels of e-selectin, intracellular adhesion molecule type 1 (ICAM-1), vascular cellular adhesion molecule-1 (VCAM-1), enhanced expression of oxidative stress level, and increased endothelial nitric oxide synthase (eNOS) [7], alongside impaired proliferation, migration, and tubular function [8]. Along with von Willebrand factor (vWF) and chemotactic C-C motif chemokine ligand 2 (CCL2), E-selectin, ICAM-1, VCAM-1, and are established markers of vascular endothelial cell dysfunction [9]. One of the most often utilized markers of vascular injury is vWF, a polymeric glycoprotein generated only in endothelial cells and megakaryocytes. It is associated with the underlying mechanisms of diabetic vascular disease. [10]. CCL2, also known as monocyte chemoattractant protein-1 (MCP-1), is a form of senescence-associated secretory phenotype (SASP) that serves as a marker for vascular endothelial cell failure and has inflammatory properties [11]. This suggests a strong correlation between HIP and its negative effects on pregnancy, primarily due to the malfunction of umbilical vein endothelial cells [12]. Therefore, exploring the mechanisms related to hyperglycemia and umbilical vein endothelial cell dysfunction may provide a new solution for preventing and treating hyperglycemia in pregnancy and other pregnancy complications and adverse pregnancy outcomes caused by it.

Recent research has demonstrated that hyperglycemia can cause endothelial cell senescence in a variety of ways. Cellular senescence is characterized by a cell’s permanent halt in growth, induced by several events such as oxidative stress, DNA damage, mitochondrial dysfunction, and oncogene activation [13,14]. Senescent cells not only exhibited the typical elevated levels of SASP and senescence-associated beta-galactosidase (SA-β-gal), but also increased the levels of the protein BCL2 that prevents apoptosis and the traditional signaling pathway proteins p53, p16^INK4A^, p21^CIP1^, and others. The core program of SASP mainly consists of interleukin-8 (IL-8), interleukin-6 (IL-6) and CCL2 [15]. Pamela’s recent research revealed a notable increase in cell cycle regulator proteins p53, p21, p16, and SA-β-gal in the human umbilical vein endothelial cells (HUVECs) of pregnant women with gestational diabetes mellitus, as opposed to typical pregnant women [16]. This indicates that hyperglycemia may induce excessive senescence of umbilical vein endothelial cells in pregnant women with HIP.

Further research has revealed that elevated blood glucose levels can promote endothelial cell dysfunction and a variety of vascular problems by causing endothelial cell senescence [17,18]. Therefore, we chose HUVECs as a model to study placental vascular senescence in pregnant women with HIP [16] and investigated the potential roles and mechanisms of hyperglycemic milieu and senescence in placental vascular function of HIP pregnant women in vitro.

## 2. Materials and Methods

### 2.1. Study Participants

The study selected HIP patients (*n* = 9) and normal pregnant women (*n* = 6) who gave birth during routine labor examinations in the Department of Obstetrics and Gynecology of the Shanghai Sixth People’s Hospital Affiliated to Shanghai Jiao Tong University School of Medicine from February 2023 to April 2023 as the study objects. Requirements for inclusion were as follows: 1. Pregestational diabetes concomitant with pregnancy: this category included individuals who had been diagnosed with type 2 diabetes before becoming pregnant; 2. Pre-diabetes: encompassing both impaired fasting glucose (IFG) and impaired glucose tolerance (IGT); 3. GDM: As per the guidelines set by the International Association of Diabetes and Pregnancy Study Groups (IADPSG) and the American Diabetes Association (ADA), Gestational Diabetes Mellitus was characterized as a condition that impacted women who had been diagnosed with GDM and had undergone a 75 g oral glucose tolerance test (OGTT) during weeks 24 to 28 of pregnancy. Individuals with type 1 diabetes, eclampsia, multiple pregnancies, and other endocrine problems were not accepted. The Ethics Committee of the Shanghai Sixth People’s Hospital, which is affiliated with the Shanghai Jiao Tong University School of Medicine, granted approval for our research. Additionally, all participants provided their informed consent by signing the necessary documents.

### 2.2. Isolation of Endothelial Cells and Experimental Design

As soon as the baby is delivered, the umbilical cord is removed and sent to a laboratory in ice. A 15 cm umbilical cord can yield roughly 40,000 HUVECs when the “perfusion–digestion” method with 0.1% collagenase type I (ThermoFisher, Waltham, MA, USA, 17018029) is used to create the cells. Following their overnight seeding in 6-well plates (with roughly 1.2 × 10^4^ cells per well), the collected HUVEC cells were replaced with new media. After being suspended in the media, the cells were overgrown and digested by pancreatic enzymes before being uniformly seeded with around 1000 cells per well in 96-well plates. The expression of the endothelial cell marker vWF (Abcam, Cambridge, United Kingdom, ab154193) was measured by immunofluorescence methods [19]. The Chinese Academy of Sciences Cell Bank (Shanghai, China) provided the HUVEC cell lines. Both primary HUVECs and HUVEC cell lines were cultivated using an endothelial cell medium (ScienCell, Santiago de Chile, CA, USA, 1001) within a cell incubator at 37 °C and 5% CO_2_. D-glucose (SIGMA, St. Louis, MO, USA, G8270-100G) is added to the complete medium until the final glucose concentration is 25 mmol/L in the high glucose medium. Hydrogen peroxide medium is created by gradually adding hydrogen peroxide to the full medium until the final concentration of hydrogen peroxide is 1 mmol/L.

In order to replicate the hyperglycemia condition in humans, we further stimulated the HUVEC cell lines (Control2 Group) and HUVECs (Control1 Group, *n* = 4) of primary normal pregnant women with high glucose medium (25 mmol/L) within 48 h. This resulted in the formation of the HG2 Group and HG1 Group. Afterward, we constructed a model to simulate replicative senescence. After six generations, the Control1 Group (*n* = 6) yielded the replicative senescence Group S1. Subsequently, the S2 Group was obtained by subjecting the Control2 Group to a 48 h treatment with a hydrogen peroxide medium (1 mmol/L) in order to create a stress-induced premature senescence (SIPS) model.

The S1 + HG1 Group was created by treating S1 with a high glucose medium (25 mmol/L) after 48 h. The S2 + HG2 Group was the co-treatment group for high glucose medium (25 mmol/L) and hydrogen peroxide medium (1 mmol/L) for 48 h.

### 2.3. Immunofluorescent Detection of vWF

Each group’s cells were uniformly planted in 96-well plates. After the cells were attached to the wall, the cells were fixed with 4% paraformaldehyde. Then, they were permeabilized with 0.5% Triton-X-100 and sealed with the immune-blocking solution. The primary vWF antibody (Abcam, ab154193) was then added at a temperature of 4 °C for the duration of the night. Subsequently, the fluorescent secondary antibody (Yeasen, Shanghai, China, 33106ES60) was incubated at a temperature of 37 °C for a period of 30 min. Finally, an adequate amount of DAPI solution was applied, and the cells were subjected to incubation at room temperature for a duration of 5 min. Finally, the cells were captured using an inverted fluorescence microscope (Olympus-IX73, Olympus, Tokyo, Japan).

### 2.4. SA-β-Gal Staining

Each group’s HUVECs were uniformly planted in 6-well plates. Cells were stained using a senescence-galactosidase staining kit (Beyotime, C0602). Subsequently, the cells were captured using an inverted fluorescent microscope (Olympus-IX73).

### 2.5. Extraction of RNA and Real-Time Quantitative PCR

Each group’s HUVECs were implanted uniformly in 6-well plates with three compound holes in each group. Using the RNA extraction kit (EZBioscience, Roswell, NM, USA, B0004DP) and the reverse transcription kit (EZBioscience, A0010CGQ), total RNA was extracted and reversed once cell adhesion reached 80% confluence. The PCR system was prepared according to the qPCR kit (EZBioscience, A0012-R1), and fluorescence quantitative qRT-PCR was performed after adding samples (Table 1). In addition, β-actin was employed to provide an endogenous control to standardize the qPCR statistics.

### 2.6. Western Blot

Each group’s HUVECs were uniformly planted in 6-well plates. Once cell adhesion and fusion reached 80%, the cells were subjected to a 30 min cold treatment in lysate containing a PMSF–RIPA ratio of 1:100, resulting in cell cracking. The cellular lysate was gathered and subjected to centrifugation at a speed of 12,000 revolutions per minute for a duration of 15 min. The supernatant was gathered, and the amount of protein was determined using the BCA protein quantification kit (NCM Biotech, Suzhou, China, WB6501). Buffer was added in proportion to protein volume (buffer liquid volume = 4:1) and heated for 10 min at 100 °C. The sample was added after gluing. After the separation of distinct relative molecular mass proteins, protein electrophoresis was accomplished at 150 V. The membrane was hermetically closed using a 5% solution of skim milk at room temperature for a length of 1 h, following transmission of 400 mA for a time of 30 min. The Tris-buffered saline plus tween-20 (TBST) was used to clean the membrane three times before incubating it in a 1/1000 dilution of anti-β-Actin (Yeasen, 30102ES60), anti-P21 (Abcam, ab109520), anti-BCL2 (Abmart, Shanghai, China, T40056), anti-P16 (Abcam, ab51243), and anti-BAX (Abmart, T40051) rabbit antibodies at 4 °C overnight. On the next day, this membrane was washed a total of three times with TBST, treated with a peroxidase-labeled goat anti-Rabbit IgG Antibody (H + L) (Yeasen, 33101ES60) diluted to a concentration of 1/10,000, and incubated at room temperature for 1 h. Afterward, the membrane was washed three more times and then exposed to a chemiluminescent solution (NCM Biotech, P10300). A hypersensitive chemiluminescence imager (Amersham Imager 600) was used to expose the film. Protein levels are quantified with Image J computer software (1.53t, National Institutes of Health, Bethesda, MD, USA).

### 2.7. The Enzyme-Linked Immunosorbent Assay (ELISA) 

ELISA was used to detect the CCL2 factor in the supernatant. Each group’s HUVEC cells were uniformly planted on a 6-well culture plate and cultured for 24 h at 37 °C in an incubator with 5% CO_2_. A total of 500 μL of medium was extracted from each well at a centrifugal force of 400 times the acceleration due to gravity and subjected to centrifugation for a duration of 20 min. The liquid portion was gathered in an EP tube and preserved around −80 °C for subsequent analysis. Following that, the Human CCL2/MCP-1 ELISA Kit (MULTI SCIENCES, Hangzhou, China, EK187-AW1) was utilized as directed by the manufacturer to measure the level of CCL2 synthesis in the supernatant. A microplate spectrometer (BioTek, Winooski, VT, USA) was used to determine the ideal density value of each well at 450 and 570 nm. Subsequently, by utilizing cytokine concentrations provided by the manufacturers as a point of reference, standard curves were generated. The average optimal density values were subsequently transformed into sample concentrations using the standard curves.

### 2.8. The CCK8 Viability Assay 

Cells in each group were uniformly planted on 96-well culture plates, and a 0.1% CCK8 solution was applied to the cells (Beyotime, C0038). After adhesion, a multifunctional enzyme labeler (Bio-Tek-Synergy H1) was used to quantify the density of each well at 450 nm after 1 h of incubation in a carbon dioxide incubator with a concentration of 5% CO_2_, maintained at a temperature of 37 °C. The proliferation capacity index is calculated as follows: experimental OD450 nm/control OD450 nm × 100%.

### 2.9. Tube Formation Assay/Matrigel Assay

A total of Fifty μL of undiluted Matrigel (Corning, New York, NY, USA, 356234) was added into each well of a pre-cooled 96-well culture plate. The plate was gently agitated until the Matrigel was well distributed. The plate was incubated at 37 °C with 5% CO_2_ for 1 h. The HUVECs in each group were treated with 0.25% EDTA trypsin, then separated by centrifugation and numbered. Approximately 4 × 10^3^ cells/mL (50 μL) were then deposited on the Matrigel-coated plate, with three compound pores inserted in each group. They were viewed and photographed using an inverted fluorescent microscope (Olympus-IX73) after incubating for 4–6 h.

### 2.10. Statistical Analysis

The statistical analysis was conducted with GraphPad Prism version 9. The results were presented as mean ± SEM. The statistical significance (*p* < 0.05) was determined using a one-way analysis of variance (ANOVA), the Student’s *t*-test, or chi-squared test.

## 3. Results

### 3.1. Clinical Characteristics of Study Participants

HUVECs extracted from normal pregnant women’s umbilical cords were assigned to the Control Group, while HUVECs extracted from HIP pregnant women were assigned to the HIP Group. Table 2 presents clinical data for HIP women (*n* = 9) and Control women (*n* = 6). The results showed no significant differences between the HIP pregnant women group and the normal pregnant women group in terms of pre-pregnancy body mass index (BMI), age, gestational week, pregnancy weight, neonatal body mass, and glycosylated hemoglobin. Nevertheless, the levels of glycated albumin in pregnant women with hyperglycemia were much greater compared to pregnant women without hyperglycemia. Furthermore, the levels of FPG, 1hPG, and 2hPG in patients with hyperglycemia were considerably greater compared to those in the Control women group.

### 3.2. Excessive Senescence of HUVECs in HIP Pregnant Women

**Figure 1:**
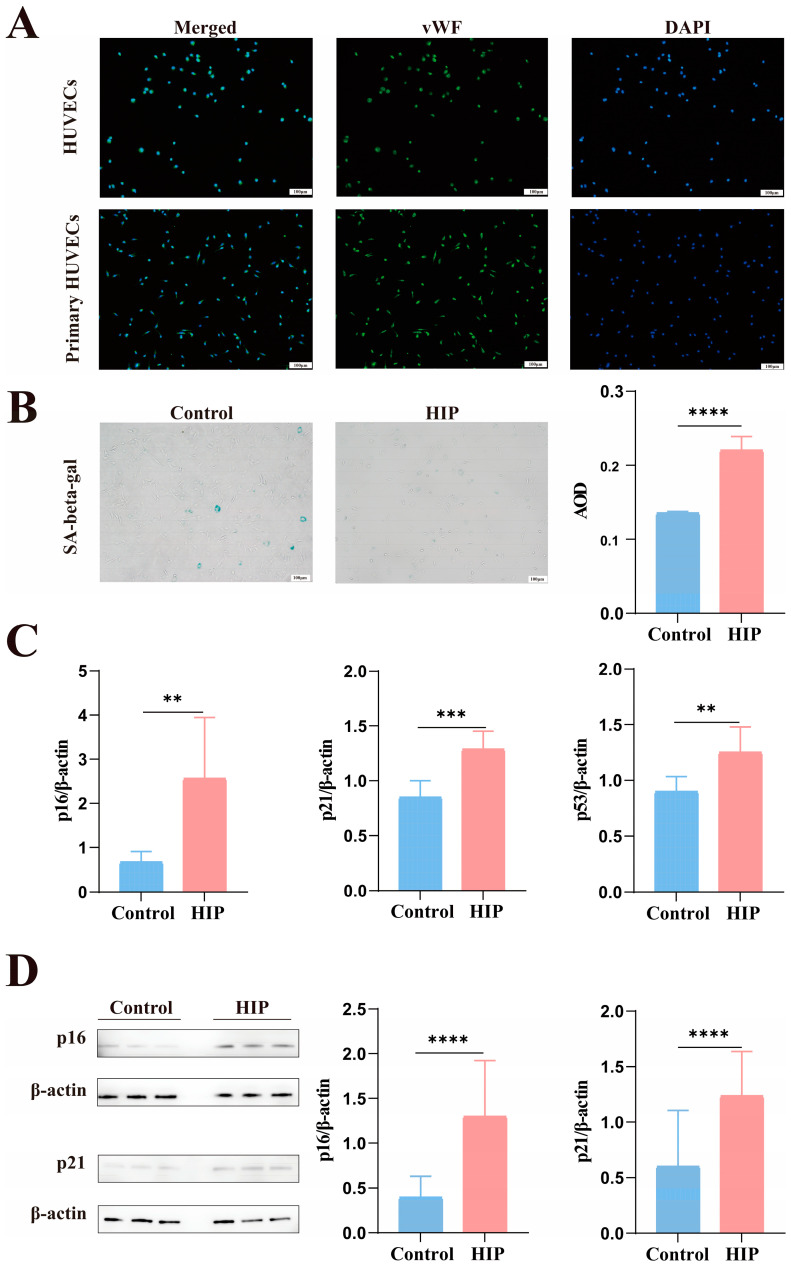
Compared to normal women, the HUVECs of HIP women overexpressed the indicators of senescence. (**A**) A cellular immunofluorescence test was utilized to identify the endothelial phenotype of HUVEC using the vWF marker. Scale bars: 100 μm. (**B**) The average optical density (AOD) statistics of HUVECs dyed with SA-β-gal staining kits in HIP women (*n* = 9) and normal women (*n* = 6). Bars of scale: 100 μm. (**C**) In comparison to the normal women, the mRNA levels of HUVECs’ p53, p16, and p21 were overexpressed in the HIP women. (**D**) The HIP Group had higher p16 and p21 protein levels. The data are shown as mean ± SEM. The Student’s *t*-test was used to determine statistical significance. ** *p* < 0.01, *** *p* < 0.001, **** *p* < 0.0001. Original figures can be found in Supplementary Materials.

First, we verified that the endothelial marker vWF was expressed in primary HUVECs and that the cell extraction was correct (Figure 1A). SA-β-gal expression in HUVECs was assessed by performing SA-β-gal staining. This analysis showed a significant rise in HUVECs in HIP pregnant women (Figure 1B). We also evaluated other aging markers, p16, p21, and p53, using qPCR and Western blot, and discovered that they were raised in both mRNA and protein levels in HIP pregnant women (Figure 1C,D).

### 3.3. High Glucose Promotes Excessive Senescence in HUVECs 

**Figure 2:**
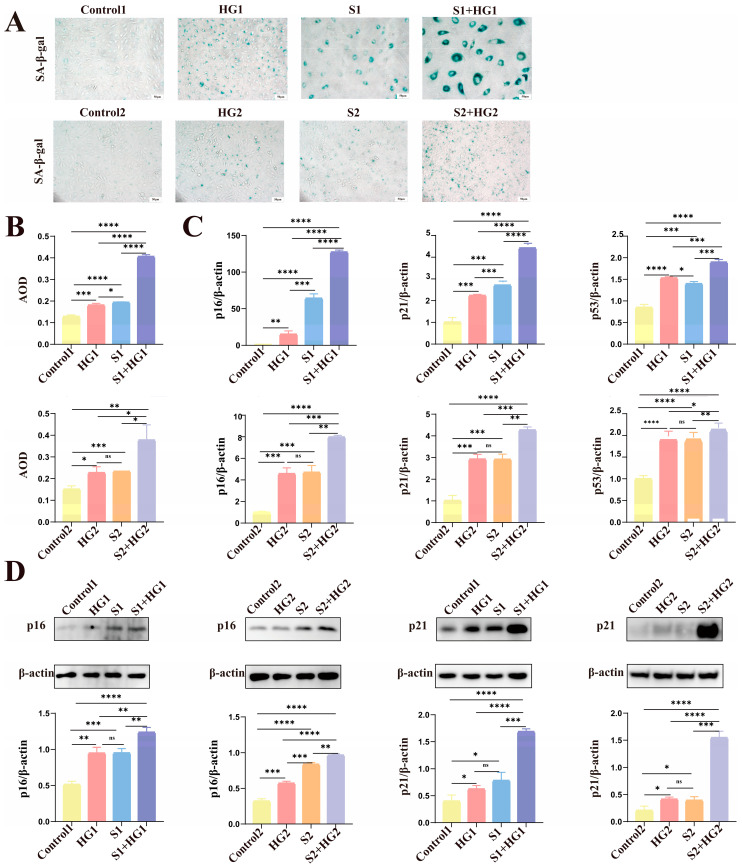
Elevated glucose levels exacerbate the senescence markers of the senescence Group and stimulate their expression in the Control Group. (**A**) SA-β-gal staining representative picture. Scale bars: 50 μm. (**B**) Statistical quantification of the average optical density (AOD) of SA-β-gal-positive cells. (**C**) The amounts of mRNA for p16, p21, and p53 were measured in each group. (**D**) The protein levels of p16 and p21 in each group. The data are represented as mean ± SEM, ns: no significant difference, * *p* < 0.05, ** *p* < 0.01, *** *p* < 0.001, **** *p* < 0.0001. Original figures can be found in Supplementary Materials.

First, we found that treating primary HUVECs and cell lines with high glucose increased levels of senescence indicators, including p53, p16, p21 (Figure 2C,D), and SA-β-gal (Figure 2A,B), which was consistent with what was reported in HIP pregnant women. Then, we constructed two senescence models: replicative senescence and SIPS, because current research has not disclosed the type of senescence observed in HUVECs generated by high glucose [20,21]. We confirmed that the primary HUVEC cells passed down to six generations and the HUVECs treated with 1 mmol/L hydrogen peroxide for 48 h achieved replicative senescence and SIPS, respectively, by analyzing levels of senescence indicators p53, p16, p21 (Figure 2C,D), and SA-β-gal (Figure 2A,B) in the senescence models. Following this, we discovered that, in addition to generating HUVEC senescence, excessive glucose increased HUVEC replicative senescence and intensified HUVEC senescence when combined with the aging inducer hydrogen peroxide (Figure 2). These results imply that high glucose not only increases HUVEC senescence but also exacerbates senescence in HUVECs that are already senescent, whether replicative senescence or SIPS.

### 3.4. High Glucose Induces Anti-Apoptotic Characteristics of Senescent HUVECs with Lower Proliferative Activity and Tube-Forming Capacity

**Figure 3:**
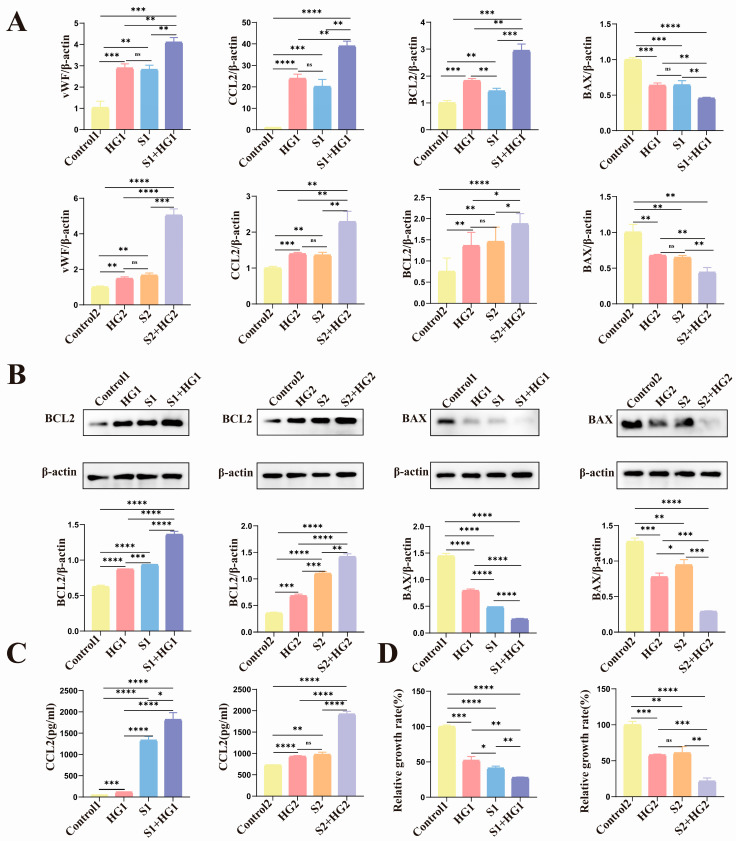
Elevated blood glucose levels cause normal and senescent HUVECs to produce more vascular damage markers, which reduces their capacity to form tubes and proliferate. (**A**) The vWF, CCL2, BCL2, and BAX mRNA levels within every group. (**B**) The protein levels of BCL2 and BAX within every group. (**C**) The amounts of CCL2 protein in each group’s supernatants. (**D**) Utilizing the CCK-8 assay, HUVECs’ cell viability was assessed. Data are represented as mean ± SEM, ns: no significant difference, * *p* < 0.05, ** *p* < 0.01, *** *p* < 0.001, **** *p* < 0.0001. Original figures can be found in Supplementary Materials.

First, our study discovered that in HUVECs subjected to elevated glucose levels and senescence, there was an increase in the expression of the anti-apoptotic molecule BCL2, whereas the production of the pro-apoptotic molecule BAX was reduced (Figure 3A,B). In both models of senescence, increasing glucose levels exacerbated the problem.

Second, we used the ELISA method to assess the secretion of CCL2, a vascular damage marker, and discovered that CCL2 expression was raised in HUVECs treated with hyperglycemia and senescence, while high glucose increased CCL2 expression in aging HUVECs (Figure 3A,C).

**Figure 4:**
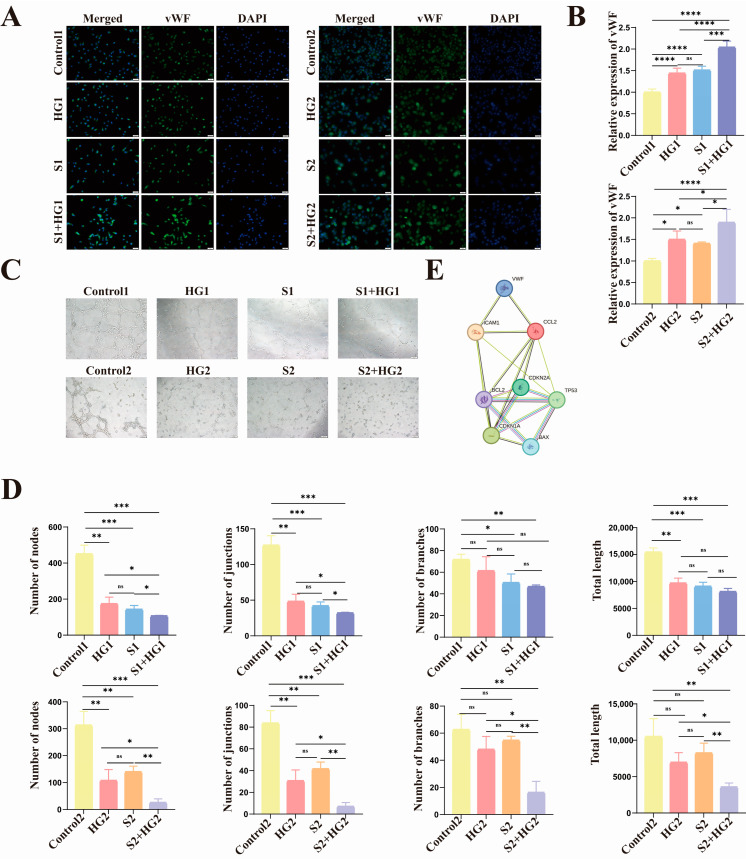
High hyperglycemia causes and exacerbates poor vascular function in HUVECs. (**A**,**B**) The protein levels of vWF are largest in the S + HG Group, subsequent to the high glucose and senescence Groups, and lowest in the Control Group. Scale = 50 μm. (**C**) Photographs of HUVEC tube development state in various circumstances. Scale = 100 μm. (**D**) Statistical quantification of the number of HUVEC nodes, junctions, branches, and total length. (**E**) P16, p21, p53, vWF, CCL2, ICAM-1, BCL2, and BAX protein-protein interactions. Data are presented as mean ± SEM, * *p* < 0.05, ** *p* < 0.01, *** *p* < 0.001 **** *p* < 0.0001, ns: no significant difference.

Following that, we discovered that both high glucose and senescence reduce HUVEC proliferation and that high glucose can synergistically suppress cell proliferation with aging (Figure 3D). To study the impact of hyperglycemia and aging on HUVEC tubule function, we added another marker of vascular injury, vWF, ICAM-1, and performed tubular studies. The results revealed that the expression trends of the three vascular damage indicators (Figure 4A,B and Figure S1A,B) were consistent, as were the trends of tube-forming ability and proliferative ability (Figure 4C,D).

Finally, as shown in Figure 4E, we used the protein interaction network to examine the interactions between p16, p21, p53, BAX, BCL2, vWF, CCL2, and ICAM-1 molecules. In general, we discovered that high glucose not only caused damage to normal vascular endothelial cells, impaired their proliferative and tube-forming ability, and inhibited apoptosis in primary HUVECs and HUVEC cell lines, but could also aggravate the damage of aging HUVECs in both replicative aging models and SIPS aging models. It exacerbated the damage to proliferative and tubule-forming abilities while inhibiting apoptosis.

### 3.5. HIP Women’s Umbilical Vein Endothelial Cells Have Anti-Apoptotic Characteristics as Well as Markedly Reduced Proliferative Activity and Tube-Forming Capacity

**Figure 5:**
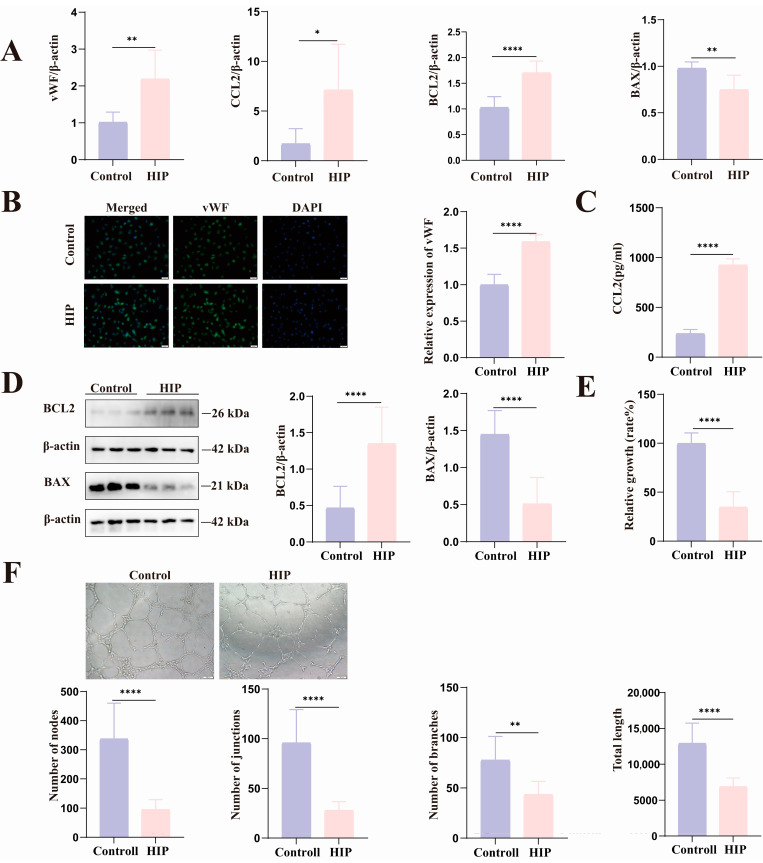
The expression of vascular damage indicators and anti-apoptotic vascular molecules increased in HIP pregnant women, but tube formation and proliferation abilities decreased. (**A**) HIP Groups had increased mRNA levels of vWF, CCL2, and BCL2, but lower levels of BAX. (**B**) vWF protein expression was increased in HIP women. (**C**) CCL2 protein levels in the supernatant were greater in the HIP Group. (**D**) BCL2 protein levels were higher in HIP Groups, whereas BAX was the opposite. (**E**) The cell viability of HIP women was compromised. (**F**) Photographs depicting the tube production stage and its statistical quantification in both the Control and HIP Groups of HUVECs. Scale = 50 μm. Data are presented as mean ± SEM, * *p* < 0.05, ** *p* < 0.01, **** *p* < 0.0001. Original figures can be found in Supplementary Materials.

Consistent with the foregoing experimental findings, the expression of vWF (Figure 5A,B), CCL2 (Figure 5A,C), ICAM-1 (Supplementary Figure S1C), and the anti-apoptotic molecule BCL2 rose in the HIP Group, whereas the pro-apoptotic protein BAX’s levels decreased (Figure 5A,D). HUVEC proliferation capacity and tubulogenesis ability were lower in HIP pregnant women than in normal pregnant women (Figure 5E,F).

## 4. Discussion

**Figure 6:**
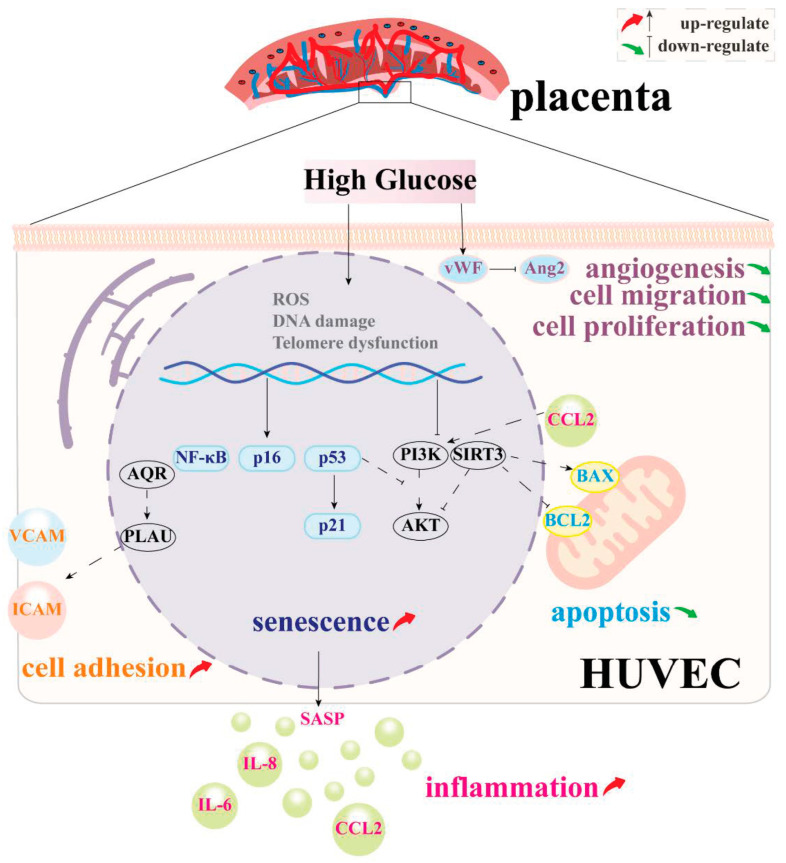
High glucose levels may cause vascular endothelial cell dysfunction by causing cell senescence. High glucose activates the p16, p53/p21, NF-κB, and AQR/PLAU pathways while inhibiting the SIRT3 and PI3K/AKT pathways, causing cell senescence and anti-apoptosis. High glucose levels can stimulate the production of adhesion molecules ICAM and VCAM via activating the AQR/PLAU pathway, as well as directly up-regulate the expression of vWF, a sign of endothelial cell injury, which promotes cell tubulogenesis, migration, and proliferation. vWF: von Willebrand factor; Ang2: angiopoietin 2; CCL2: chemotactic C-C motif chemokine ligand 2; BCL2: B-cell lymphoma 2; SIRT3: sirtuin 3; ICAM: intracellular adhesion molecule type; VCAM: vascular cellular adhesion molecule; IL-8: interleukin-8; IL-6: interleukin-6; SASP: senescence-associated secretory phenotype.

Our study indicated that high glucose accelerates and aggravates vascular endothelial cell aging, aligning with previous findings [16]. Hyperglycemia can cause the cessation of the cell cycle in endothelial cells through the action of cyclin-dependent kinase inhibitors, specifically p21^CIP1^ and p16^INK4A^ [22], resulting in accelerated anti-senescence of endothelial cells. The activation of the DNA damage response (DDR) pathway is triggered by many senescence factors that promote telomere dysfunction and DNA double-strand breaks (DSBs). The activation of ataxia telangiectasia mutated (ATM) and ataxia telangiectasia and Rad3-related (ATR) occurs in response to the accumulation of DSBs. The p53/p21^CIP1^ pathway [23] is triggered by the ATM-CHK2/ATR-CHK1 (checkpoint kinase) pathway [24], which enhances the synthesis of the p21^CIP1^ protein, leading to cellular senescence. The DNA damage response pathway can also trigger the activation of p16^INK4A^, which hinders the function of cyclin-dependent kinases 4 and 6 (CDK4/6) and causes the accumulation of phosphorylated pRB. Consequently, this contributes to the termination of transcription factor E2F regulation and ultimately results in cell cycle arrest [24]. Besides, hyperglycemia can cause DNA damage and activate p53 [25,26] by producing methylglyoxal, as well as decrease the expression of sirtuin 3 (SIRT3) [17] and sirtuin 1 (SIRT1) [27] in HUVECs and promote HUVEC senescence via AQR/PLAU-related pathways [18] (Figure 6).

Additionally, our research found that endothelial cell dysfunction, as well as tubular and proliferative abilities, were compromised, and that anti-apoptosis was mostly driven by HUVEC senescence caused by high glucose levels. However, the mechanism by which high-glucose-induced senescence of HUVECs leads to dysfunction is still unknown. Previous research reveals that senescent endothelium cells produced by high glucose levels may cause endothelial cell dysfunction in a variety of ways. First, high glucose levels raise the production of p53, a critical protein in cell cycle regulation that adversely inhibits eNOS phosphorylation and impels endothelial activity via the p53/PTEN/eNOS pathway [28]. Second, excessive glucose levels stimulate AOR gene overexpression, which disrupts the cell cycle and causes cell aging. Overexpressed AQR then stimulates the AQR/PLAU pathway, increasing inflammatory factor release and elevated levels of vascular adhesion molecules such as VCAM1 and ICAM1, resulting in vascular endothelial cell dysfunction [18]. Third, excessive glucose levels suppress the anti-aging molecule SIRT3. SIRT3 not only protects cells from angiogenesis diseases, but it also possesses anti-aging properties. Reduced SIRT3 expression significantly reduces HUVECs’ ability to form capillary-like networks [17]. Fourth, elevated glucose levels inhibit the PI3K/AKT pathway, compromising cells’ capacity to handle endoplasmic reticulum stress, cellular damage, reactive oxygen species (ROS), and inflammatory factors induced by high glucose [29,30] (Figure 6).

High glucose levels have been proven in the literature to trigger apoptosis [31], although it has also been shown that aged cells have anti-apoptotic capabilities [32]. Our findings suggest that anti-apoptosis appears to be dominant in pregnant women with HIP. Senescence cells have an anti-apoptotic function, which results in the delayed replacement of defective vascular endothelial cells by new endothelial cells with good tube forming ability and proliferation ability that can perform normal physiological functions, resulting in tubular dysfunction and proliferation of placental vascular endothelial cells, which results in placental hypertrophy, decreased material transport efficiency, and placental vascular remodeling disorders [33]. The normal progression and maturation of the fetus, together with the well-being of the mother, are significantly impeded.

This study adds to the data that excessive glucose levels in HIP pregnant women increase placental vascular senescence. There are currently few relevant research using HUVECs taken from HIP pregnant women as a model to study endothelial cell senescence induced by high glucose, and our study is a helpful addition to the deficiencies in this respect. In addition, our study found that high glucose levels also caused senescence of placental vascular endothelium cells in HIP pregnant women, causing placental vascular dysfunction. Investigating the mechanisms of pregnancy problems and bad pregnancy outcomes in HIP pregnant women yields novel insights. However, this study does have certain limitations. First, in terms of research methodologies, the narrow selection sample range is limited, and an insufficient sample size may impair the accuracy of experimental results. Second, the research content focuses primarily on the impact of endothelial cell senescence induced by high glucose on its dysfunction, which leads to the occurrence of pregnancy complications and negative pregnancy outcomes, but the impact of anti-aging and anti-glucose drugs on this process is not addressed.

Despite the fact that we have many classic anti-senescence medications, metformin, rapa penicillin, and aerosol are the most commonly used [34,35]. However, there is no study to indicate whether anti-senescence drugs have the same effect on endothelial cell dysfunction caused by delayed aging. New research, however, suggests that metformin may help to prevent endothelial cell dysfunction induced by senescence [36,37]. Metformin can activate the anti-aging pathway AMPK and the essential molecule SIRT1 [38], as well as the PI3K/AKT/eNOS signaling pathway. This promotes vasodilation and safeguards blood vessels. Endothelial cell dysfunction caused by cell aging can be alleviated by lowering the plasma levels of vWF, sVCAM, and sICAM [39]. In addition, insulin, which also regulates blood glucose, may also alleviate aging-induced endothelial cell dysfunction [40]. In vitro, insulin can mitigate endothelial cell senescence and vascular dysfunction by diminishing the production of nitric oxide (NO) and ROS in HUVECs [41]. The theory and practice of medications used to prevent endothelial cell failure induced by cell senescence are not ideal at the moment. More research is needed to discover whether it is clinically feasible.

## 5. Conclusions

It can be concluded that elevated glucose levels cause senescence of placental vascular endothelial cells. Senescent endothelial cells not only have decreased proliferation, migration, and angiogenesis ability, but their anti-apoptotic properties also prevent the placenta from clearing the dysfunctional senescent endothelial cells in time, resulting in vascular remodeling disorders and placental malperfusion, and the placental bed cannot provide a good environment for fetal growth and development. Pregnancy problems and unfavorable pregnancy outcomes, such as eclampsia, have increased significantly [42,43].

## Figures and Tables

**Table 1:** Forward and reverse primers for target genes are provided in sequential order.

Name	Forward Primer, 5′-3′	Reverse Primer, 5′-3′
β-actin	TGGCACCCAGCACAATGAA	CTAAGTCATAGTCCGCCTAGAAGCA
p16	CATGGAGCCTTCGGCTGAC	GCGCTGCCCATCATCATG
p21	TGTCACTGTCTTGTACCCTTG	GGCGTTTGGAGTGGTAGAA
p53	GTACCACCATCCACTACAACTAC	CACAAACACGCACCTCAAAG
BCL2	TGTGGATGACTGAGTACCTGAACC	CAGCCAGGAGAAATCAAACAGAGG
BAX	AAGCGACTGATGTCCCTGTCTC	GATGGTGAGTGAGGCGGTGAG
vWf	ACCTTGGTCACATCTTCACATTCAC	GTCATTGGCTCCGTTCTCATCAC
CCL2	CAGCCAGATGCAATCAATGCC	TGGAATCCTGAACCCACTTCT
ICAM-1	ACCTATGGCAACGACTCCTTCTC	GTGTCTCCTGGCTCTGGTTCC

**Table 2:** Clinical information of normal and HIP women who are participating in the study.

Index	Control Group (*n* = 6)	HIP Group (*n* = 9)	*p* Value
Age (year)	30.17 ± 1.72	31.00 ± 4.56	0.6785
Gestational age (weeks)	39.02 ± 0.84	39.29 ± 1.53	0.7108
Pre-pregnancy BMI (kg/m^2^)	25.97 ± 2.27	27.43 ± 4.23	0.4551
Pregnancy weight gain/kg	14.30 ± 3.88	12.33 ± 3.01	0.2887
FPG/(mmol/L)	4.45 ± 0.23	5.00 ± 0.41	0.0019
1hPG/(mmol/L)	6.87 ± 1.47	9.70 ± 1.16	0.0011
2hPG/(mmol/L)	6.07 ± 0.79	8.49 ± 1.42	0.0022
Glycated albumin (%)	10.80 ± 1.07	12.99 ± 1.33	0.0051
Glycosylated hemoglobin (%)	5.22 ± 0.15	5.36 ± 0.42	0.4392
Newborn body mass (g)	3678.00 ± 513.32	3387.00 ± 573.17	0.3443

Note: The average ± standard error of the mean or a percentage (%) was used to report the results; OGTT: 75 g D-glucose oral glucose tolerance test; BMI: body mass index, BMI (kg/m^2^) = weight (kg)/height^2^ (m^2^); FPG: fasting plasma glucose; 1hPG/2hPG: 1 or 2 hours’ postprandial glucose in the OGTT, or 1 or 2 hours’ postprandial glucose.

## Data Availability

The data supporting this article will be made available to the associated author upon reasonable request.

## Supplementary Materials

The following supporting information can be downloaded at: https://www.mdpi.com/article/10.3390/biom14030329/s1, Figure S1: Normal and senescent HUVECs release more of the vascular injury marker ICAM-1 in response to elevated blood glucose levels. Original figures can be found in Supplementary Materials.
