# Supplementary material for: High Glucose Promotes and Aggravates the Senescence and Dysfunction of Vascular Endothelial Cells in Women with Hyperglycemia in Pregnancy
Source: Biomolecules. 2024 Mar 10;14(3):329. doi: 10.3390/biom14030329 (PMC10968295; doi:10.3390/biom14030329)
Supplement: Supplementary file 1 [file biomolecules-14-00329-s001.zip › biomolecules-2849767-Supplemental Figure S1.pdf]

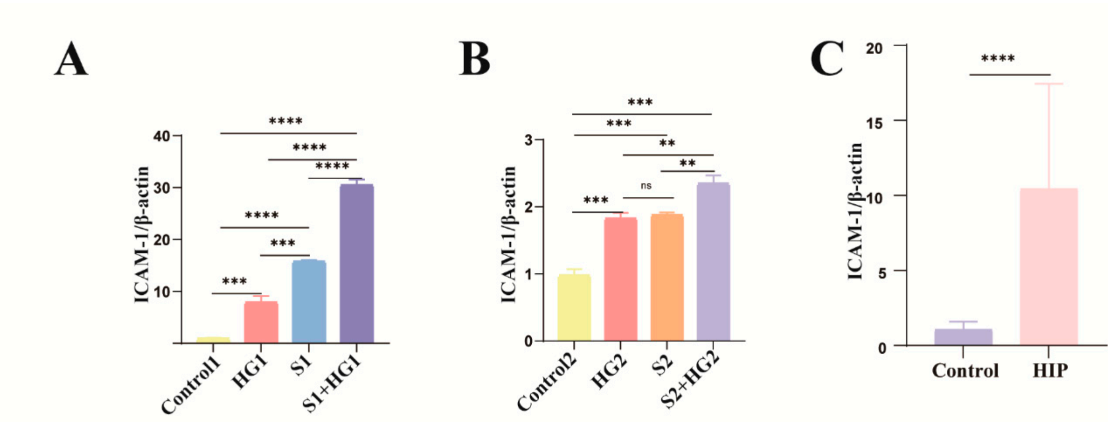

Supplemental Figure S1. Normal and senescent HUVECs release more of the vascular injury marker ICAM-1 in response to elevated blood glucose levels. ICAM-1 expression rose in HIP pregnant women. (A) (B) The amounts of ICAM-1 mRNA in each group. (C) HIP Groups exhibited higher ICAM-1 mRNA levels. Data are represented as mean ± SEM, ns: no significant difference, ∗∗P < 0.01, ∗∗∗P < 0.001, ∗∗∗∗P < 0.0001, ns: no significant difference.
