# Supplementary figures and images for: High Glucose Promotes and Aggravates the Senescence and Dysfunction of Vascular Endothelial Cells in Women with Hyperglycemia in Pregnancy
Source: Biomolecules. 2024 Mar 10;14(3):329. doi: 10.3390/biom14030329 (PMC10968295; doi:10.3390/biom14030329)

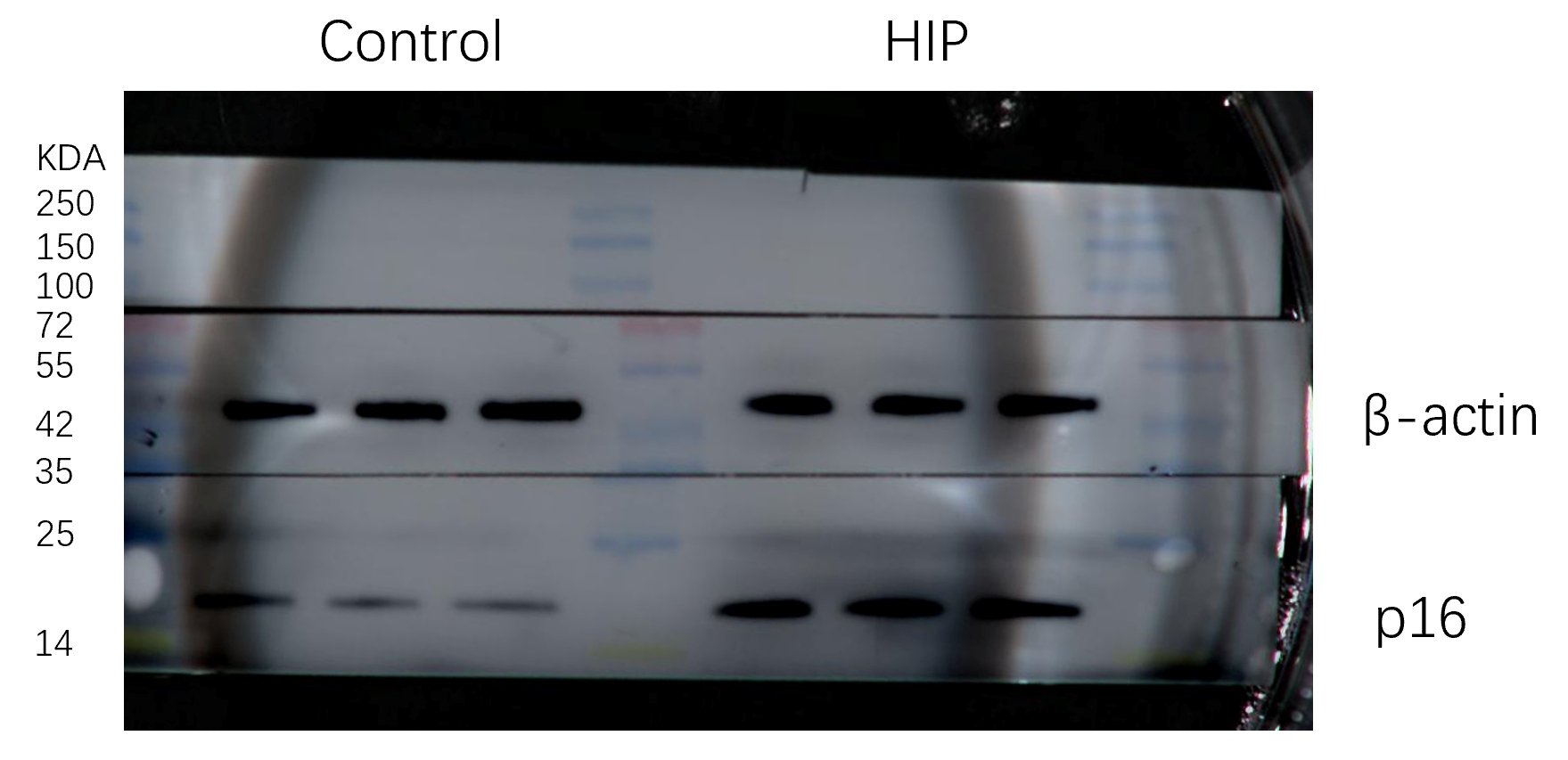

Supplement: Supplementary file 1 [file biomolecules-14-00329-s001.zip › Figure1-D-1.jpg]

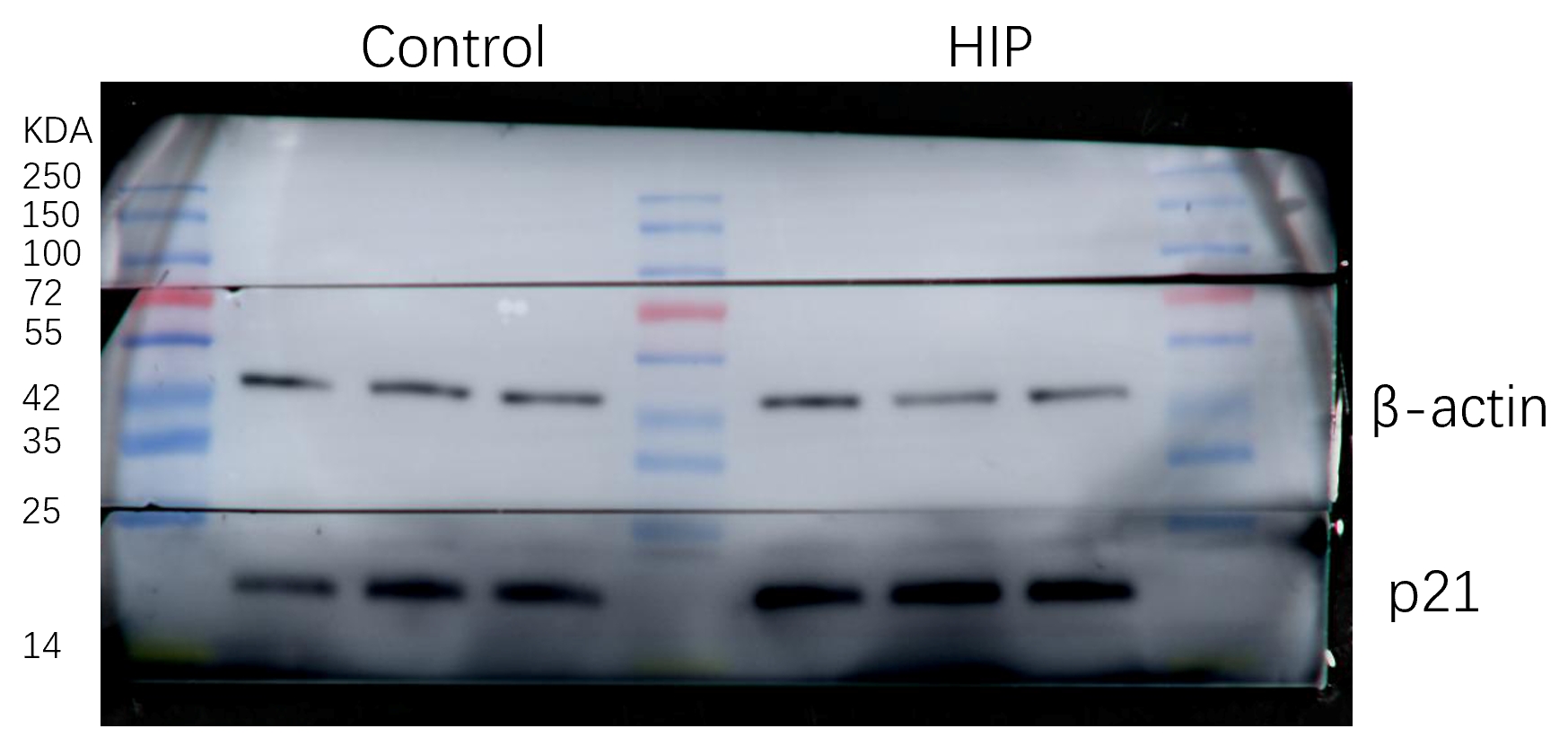

Supplement: Supplementary file 1 [file biomolecules-14-00329-s001.zip › Figure1-D-2.jpg]

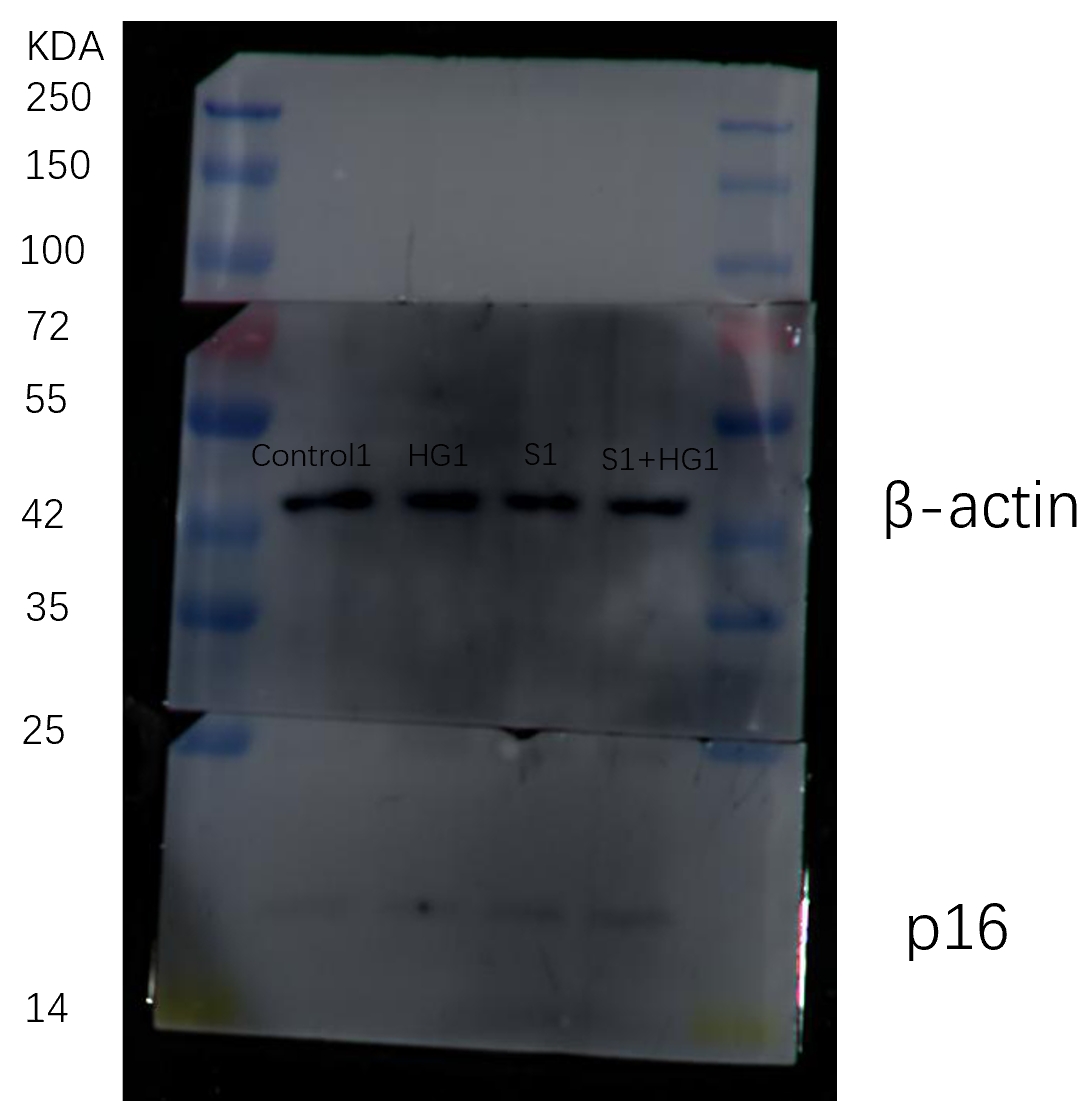

Supplement: Supplementary file 1 [file biomolecules-14-00329-s001.zip › Figure2-D-1.jpg]

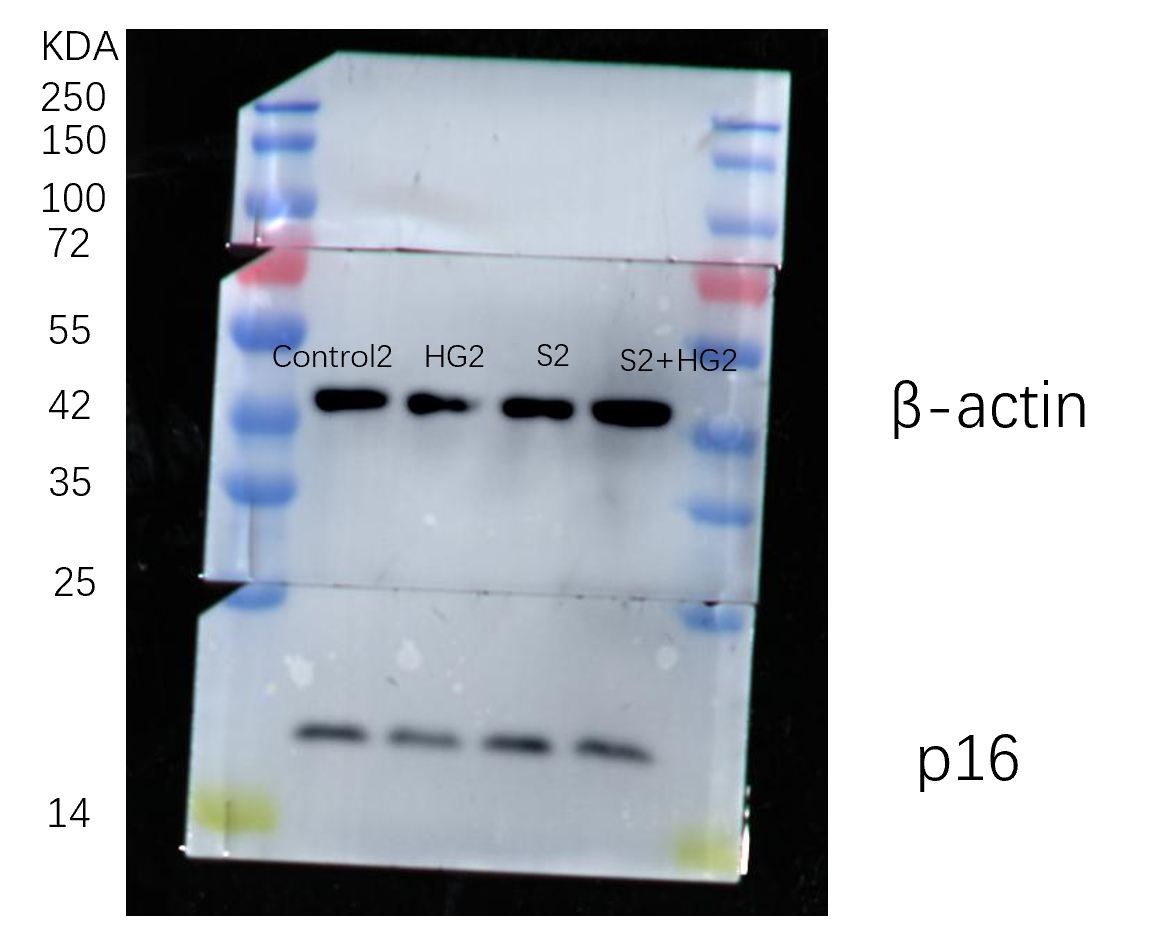

Supplement: Supplementary file 1 [file biomolecules-14-00329-s001.zip › Figure2-D-2.jpg]

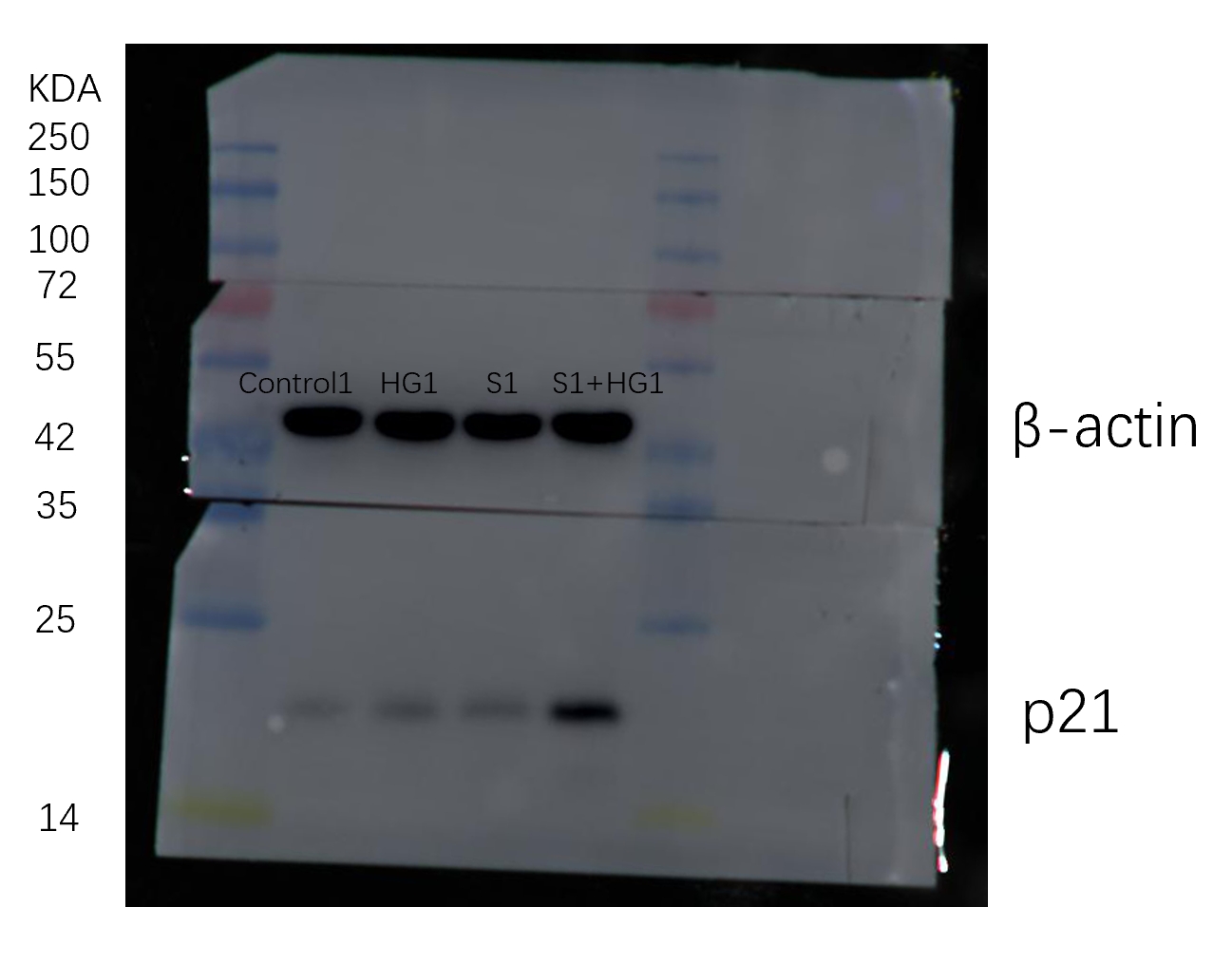

Supplement: Supplementary file 1 [file biomolecules-14-00329-s001.zip › Figure2-D-3.jpg]

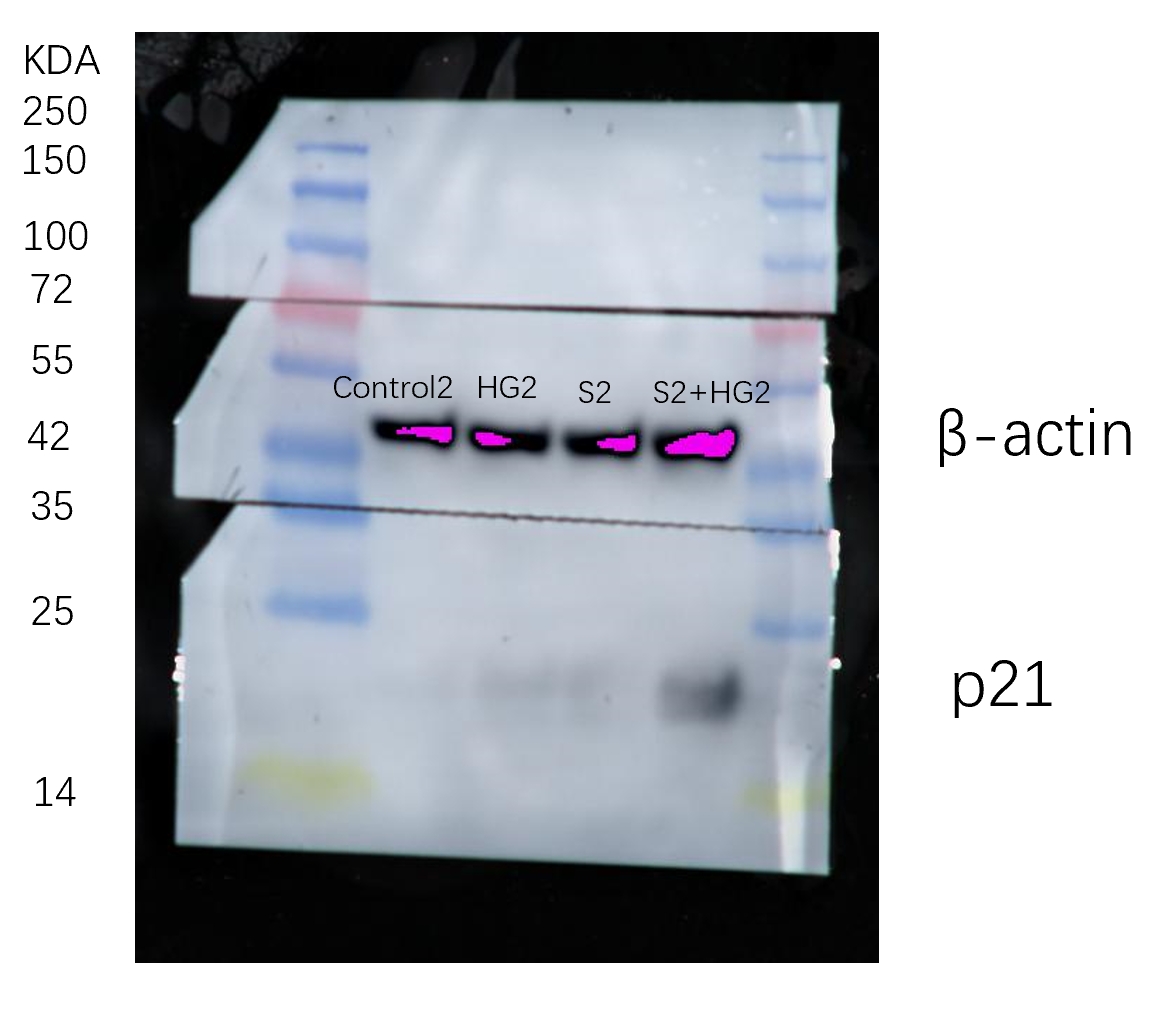

Supplement: Supplementary file 1 [file biomolecules-14-00329-s001.zip › Figure2-D-4.jpg]

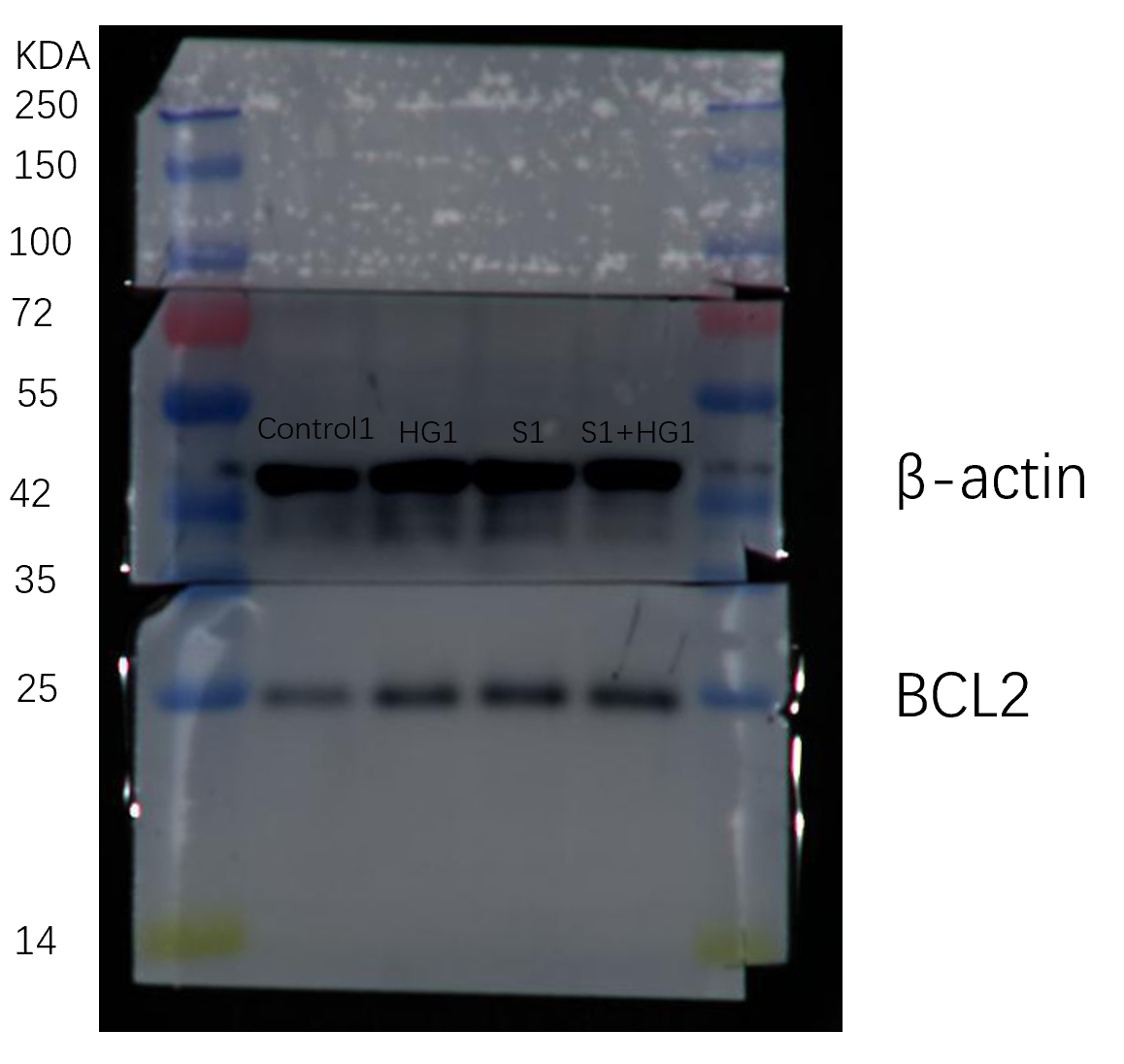

Supplement: Supplementary file 1 [file biomolecules-14-00329-s001.zip › Figure3-B-1.jpg]

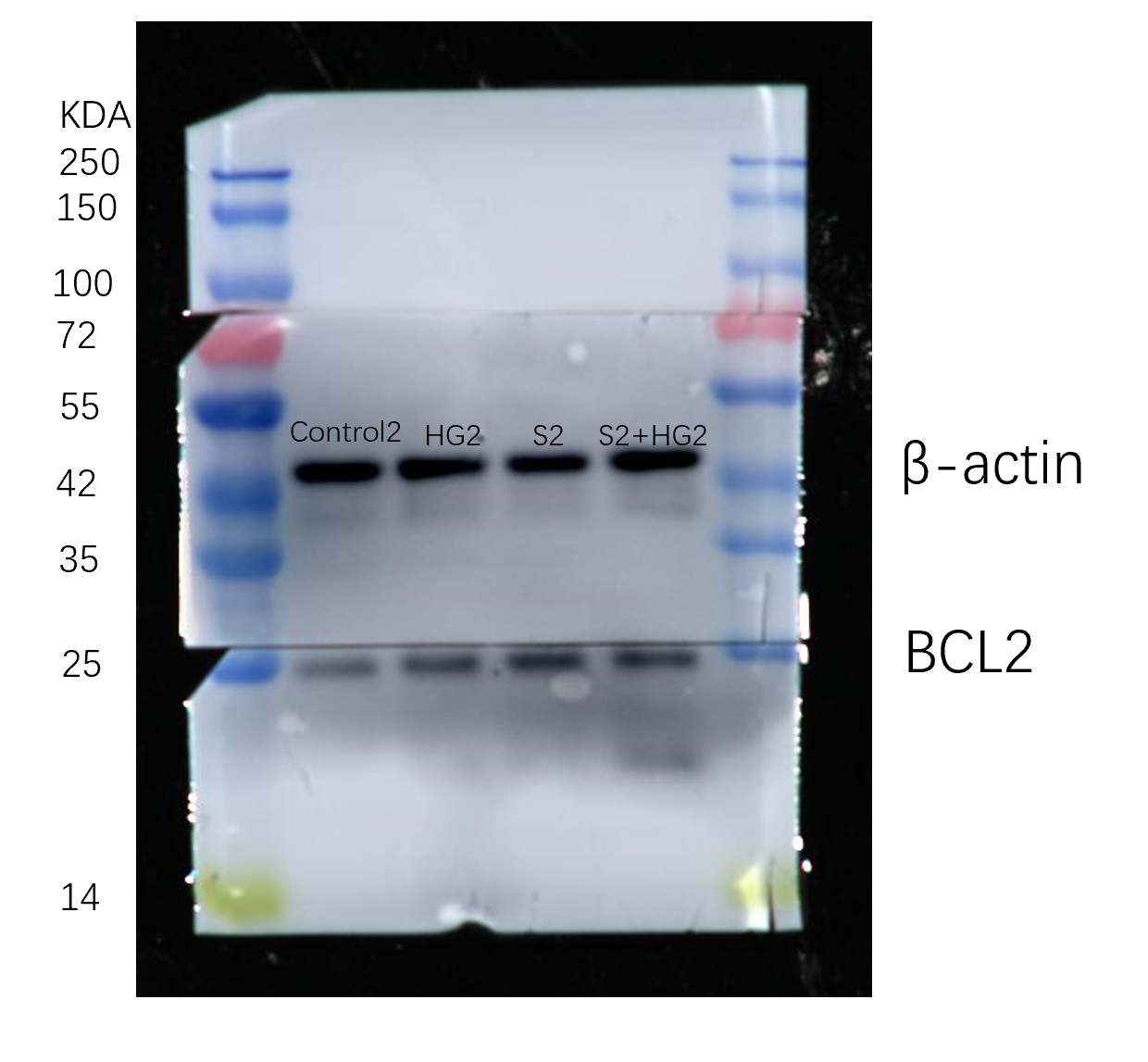

Supplement: Supplementary file 1 [file biomolecules-14-00329-s001.zip › Figure3-B-2.jpg]

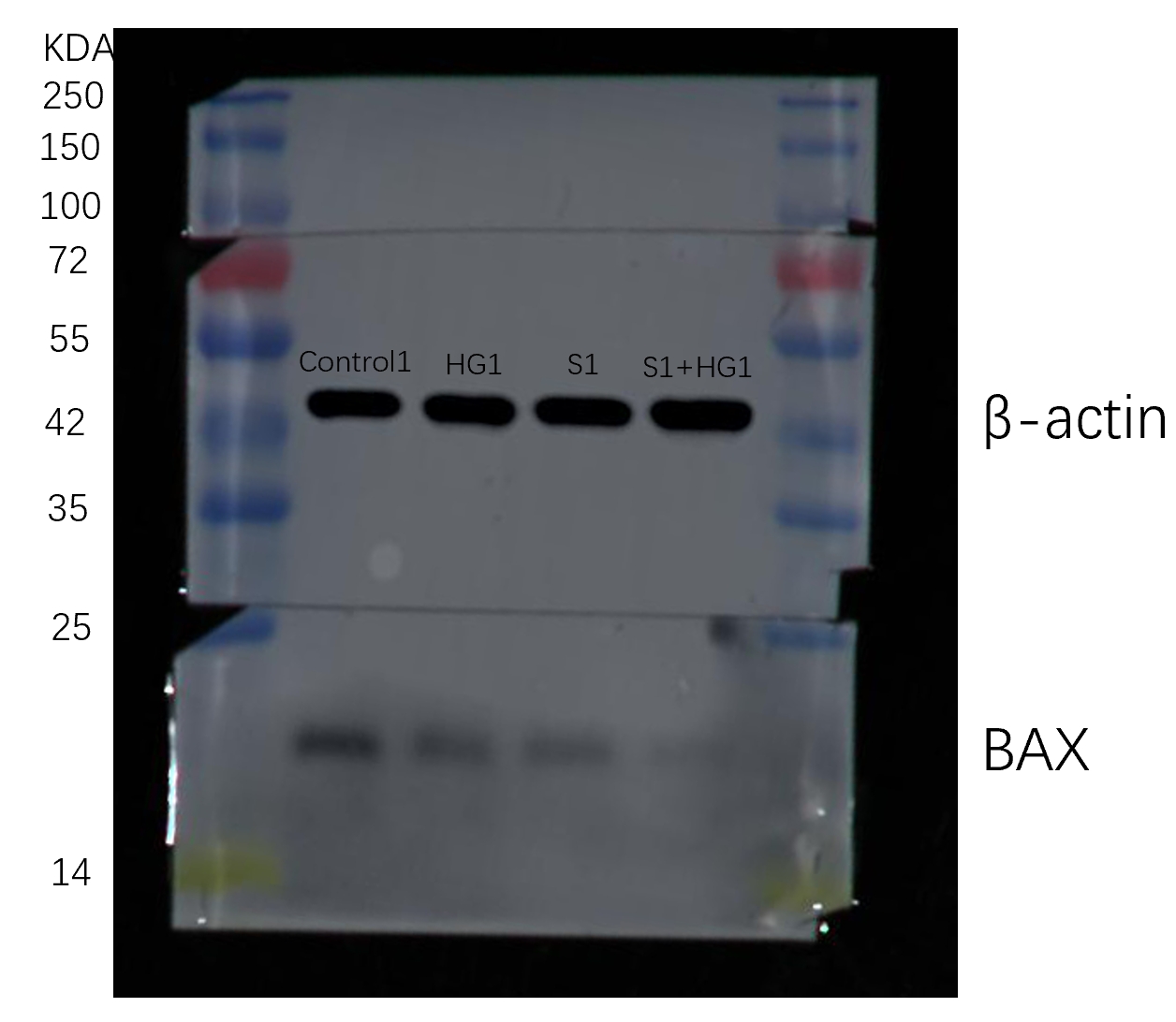

Supplement: Supplementary file 1 [file biomolecules-14-00329-s001.zip › Figure3-B-3.jpg]

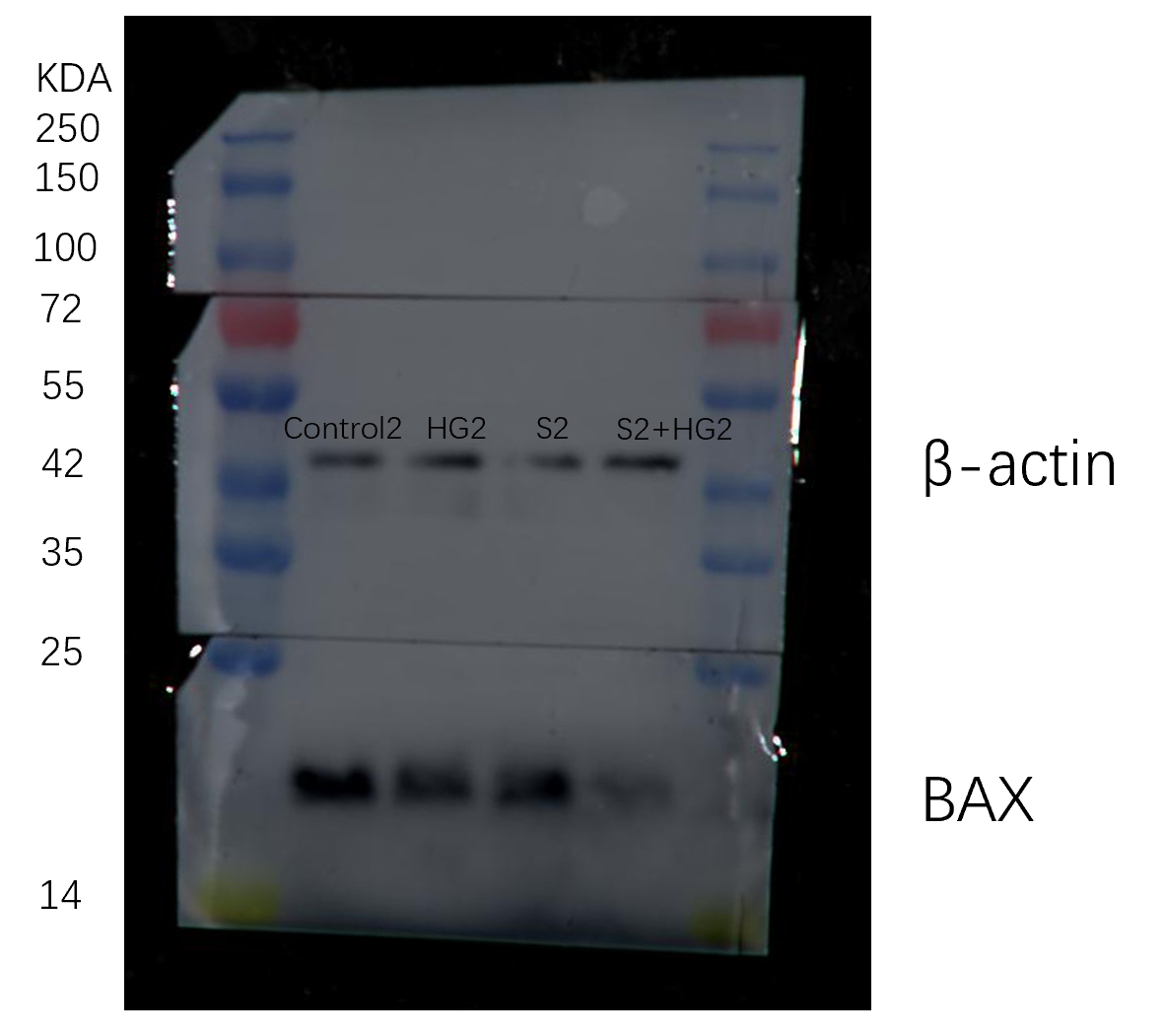

Supplement: Supplementary file 1 [file biomolecules-14-00329-s001.zip › Figure3-B-4.jpg]

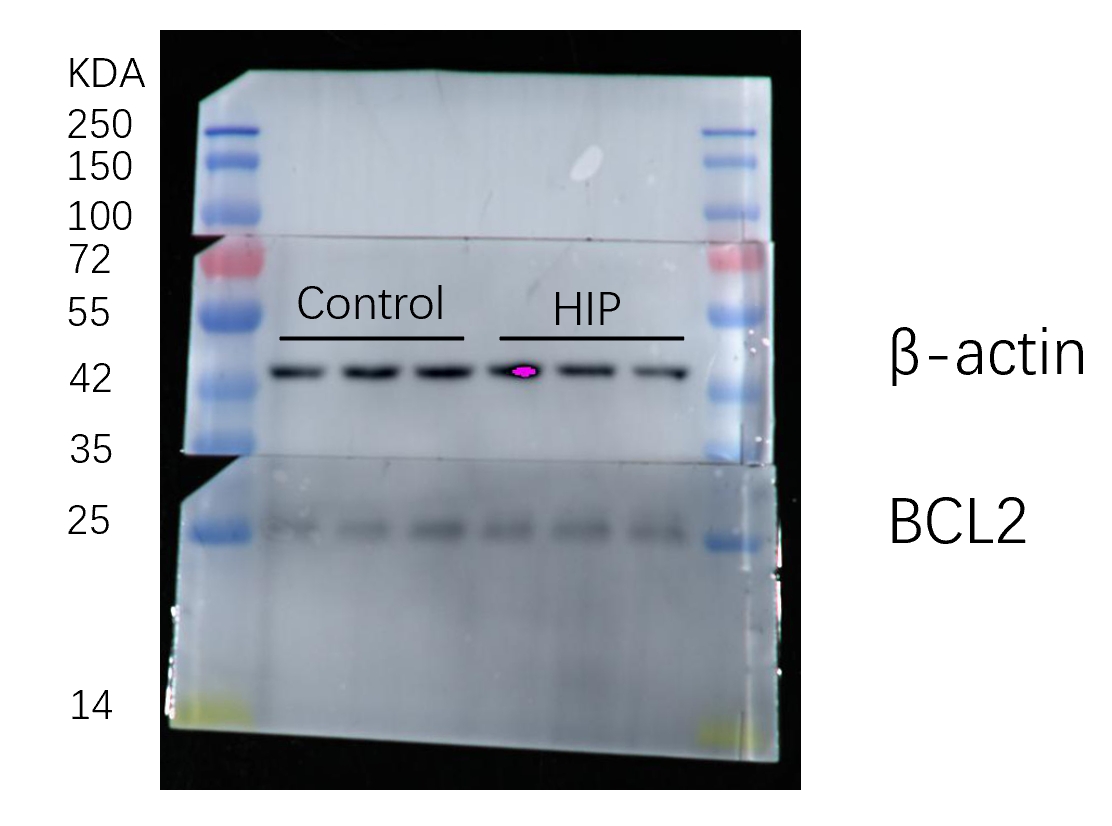

Supplement: Supplementary file 1 [file biomolecules-14-00329-s001.zip › Figure5-D-1.jpg]

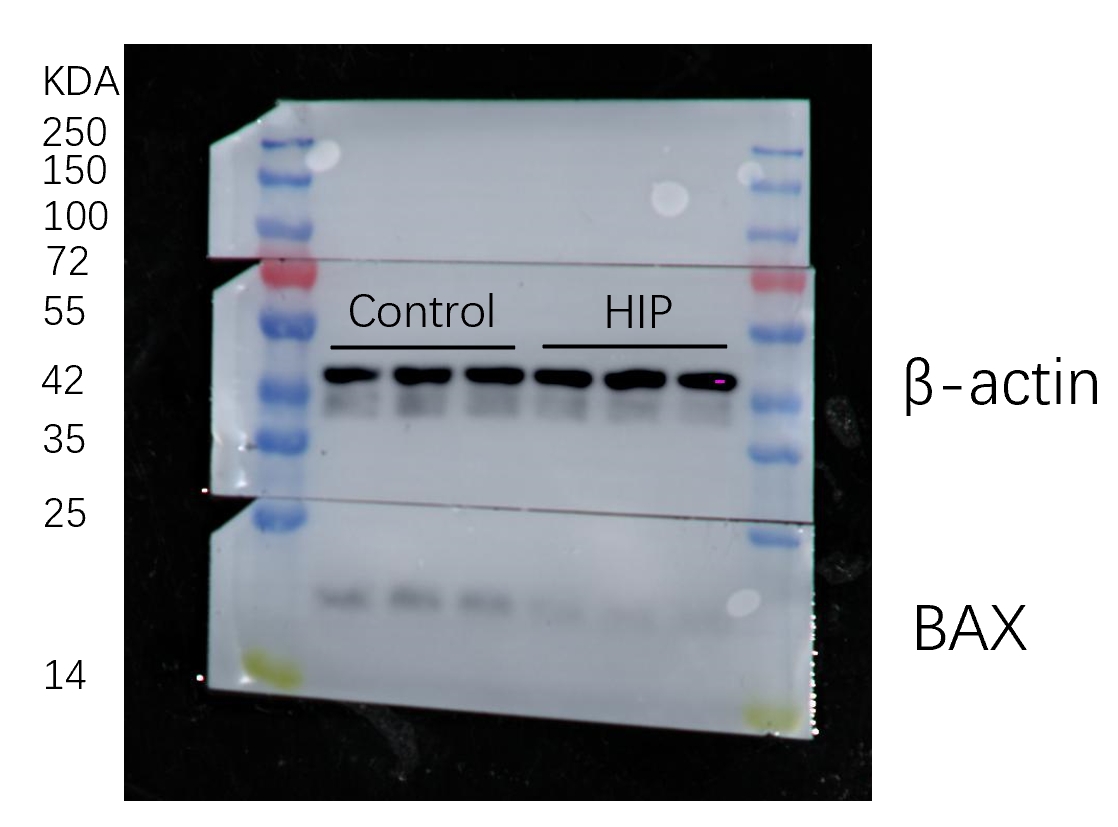

Supplement: Supplementary file 1 [file biomolecules-14-00329-s001.zip › Figure5-D-2.jpg]
